# Developing and testing a principle-based fidelity index for peer support in mental health services

**DOI:** 10.1007/s00127-021-02038-4

**Published:** 2021-02-19

**Authors:** S. Gillard, N. Banach, E. Barlow, J. Byrne, R. Foster, L. Goldsmith, J. Marks, C. McWilliam, R. Morshead, K. Stepanian, R. Turner, A. Verey, S. White

**Affiliations:** 1 Population Health Research Institute, St George’s, University of London, London, UK; 2 Research and Development Department, Sussex Partnership NHS Foundation Trust, Hove, UK; 3 School of Human and Health Sciences, University of Huddersfield, Huddersfield, UK; 4 School of Health Sciences, City, University of London, London, UK

**Keywords:** Peer support, Fidelity index, Community mental health, Randomised controlled trials, Social support, Validation study

## Abstract

**Purpose:**

Evidence suggests that the distinctive relational qualities of peer support—compared to clinical-patient relationships—can be eroded in regulated healthcare environments. Measurement of fidelity in trials of peer support is lacking. This paper reports the development and testing of a fidelity index for one-to-one peer support in mental health services, designed to assess fidelity to principles that characterise the distinctiveness of peer support.

**Methods:**

A draft index was developed using expert panels of service user researchers and people doing peer support, informed by an evidence-based, peer support principles framework. Two rounds of testing took place in 24 mental health services providing peer support in a range of settings. Fidelity was assessed through interviews with peer workers, their supervisors and people receiving peer support. Responses were tested for spread and internal consistency, independently double rated for inter-rater reliability, with feedback from interviewees and service user researchers used to refine the index.

**Results:**

A fidelity index for one-to-one peer support in mental health services was produced with good psychometric properties. Fidelity is assessed in four principle-based domains; building trusting relationships based on shared lived experience; reciprocity and mutuality; leadership, choice and control; building strengths and making connections to community.

**Conclusions:**

The index offers potential to improve the evidence base for peer support in mental health services, enabling future trials to assess fidelity of interventions to peer support principles, and service providers a means of ensuring that peer support retains its distinctive qualities as it is introduced into mental health services.

**Supplementary Information:**

The online version contains supplementary material available at 10.1007/s00127-021-02038-4.

## Background

In recent years peer support has been introduced into formal mental health services internationally, often in the form of the ‘peer worker’ role, whereby an individual with personal experience of using mental health services is trained and employed to explicitly use that experience in supporting others currently using services (their peers). It has been widely argued that peer support in mental health services is grounded in peer-to-peer relationships that are highly distinctive from clinician–patient relationships, with peer-to-peer relationships underpinned by: a sense of connection between peers based on a recognition of shared experiences [1]; reciprocity in the relationship whereby both parties learn from each other [2]; the validation and exchange of experiential, rather than professionally acquired knowledge [3]. However, research has indicated that organisational factors relating to implementation of peer support can impact on the extent to which peer workers feel able to make use of their personal experiences in supporting others [4]. It is possible that a resulting lack of distinctiveness of peer support—compared to other forms of mental health support—explains, at least in part, why reviews of peer support interventions have largely indicated that peer support is ‘no better or worse’ than similar work done by other mental health workers [5, 6]. A recent review of peer support in mental health services concludes both that good measures of fidelity of peer support interventions are needed, and that a lack of attention to fidelity to the core principles underpinning the distinctiveness of peer support limits the usefulness of current peer support research to policy makers and practitioners [7].

Treatment fidelity in healthcare outcomes research is classically defined as ‘confirmation that the manipulation of the independent variable occurred as planned’ [8], or more broadly, that a treatment is both implemented as intended, and is demonstrably differentiated from an alternative treatment condition, including treatment as usually provided. Measurement of intervention fidelity, through development and use of a scale or index, helps ensure the internal validity of outcomes research, can account for negative or ambiguous findings, enables documentation of deviation or differences in the implementation of an intervention, supports study replication and meta-analyses, and can act as a moderating variable to explain variance in outcomes [9]. Where an intervention is complex—where there are multiple active ingredients that comprise an intervention—a fidelity index should operationalise that complexity. Accordingly, it is recommended that a number of elements of fidelity should be assessed in addition to delivery of the intervention; for example, that the training and supervision of staff delivering the intervention should also be assessed as a key fidelity ingredient [9]. Psychosocial interventions in healthcare are necessarily complex as they assume that human qualities, including social or relational mechanisms—e.g., between patient and clinician—are an active ingredient of the intervention. For example, the chronic care model for managing long-term conditions [10] assumes that the ‘activated patient’ and ‘informed clinician’ are the important components of an intervention, alongside any self-management tools that might be utilised. As a result, measurement of fidelity of chronic care interventions typically assesses not only the utilisation of tools in accordance with an intervention manual but also the level of responsiveness to the intervention of both patient and clinician [11, 12].

If the distinctive qualities of peer-to-peer relationships are core to the way in which a peer support intervention works, it is therefore, not sufficient to define fidelity in terms of, for example, how often or for how long peers meet, or the extent to which they make use of manualised tools. Measurement of fidelity of peer support should also seek to assess fidelity to principles that characterise peer-to-peer relationships [13, 14]. However, measurement of fidelity in the evaluation of peer support remains the exception rather than the norm. A fidelity tool for the evaluation of peer respite services in the US [15] tested 46 items in the domains of structure, environment, belief systems, peer support, education and advocacy. The fidelity of delivery of a mental illness management and recovery programme by peer workers was compared with delivery of the same programme by mental health professionals [16], but the fidelity of peer support itself was not tested. Chinman et al. [17] note a lack of evidence offering insight into whether the absence of effect demonstrated in a number of recent trials of peer support interventions is attributable to ineffective peer support or to the peer support not having been delivered as intended. They have undertaken preliminary work to develop a measure of fidelity of delivery of peer specialist services, across various mental health settings, in two content areas; delivery of peer specialist activities, and implementation factors that support or hamper delivery [17], with early findings demonstrating appropriateness of using self-report questionnaires with peer specialists and their supervisors as an approach to testing fidelity.

Nonetheless, the focus of these tools remains largely on the delivery of intervention activity (what peer workers do). A need remains for a study that develops and tests a measure of fidelity of peer support in mental health services that explicitly assesses the extent to which fidelity to the principles that define the distinctiveness of peer support (compared to other forms of mental health support) is demonstrated.

## Method

### Study design

This paper reports a study that develops and tests a ‘principle-based’ fidelity index for one-to-one peer support in mental health services. The fidelity study was part of a larger programme of research to develop and trial peer support for discharge from inpatient to community mental health care [18]. The study follows a full cycle of fidelity index development; identifying and specifying fidelity criteria, measuring fidelity, and assessing the reliability and validity of fidelity criteria [19].

It has been recommended that the theory or framework underpinning an intervention should be specified in any manuals or guidelines for delivering the intervention, and should also inform assessment of fidelity [9]. The development of the intervention being tested here was underpinned by a ‘principles of peer support’ framework [20] designed to guide implementation of peer support values into practice. The principles were developed through systematic review of the informal and formal research literature on peer support, and consensus workshops with an expert panel that were independent from the research team. Three panel members had worked as peer workers and/or led peer support services in the National Health Service in England, two led peer support services in the not-for-profit section, and five had undertaken research about peer support and mental health, including research from a service user or survivor perspective [20].

### Item development

We held two workshops to generate items for the index, one each with the expert panel that helped develop the principles framework and with members of the multidisciplinary research team. We began by identifying fidelity criteria; statements about the peer worker role and how peer support worked in practice, whereby, agreement with the statement would signify adherence to the principles. We asked workshop members to identify criteria that applied to each of the five principles from the framework: (1) relationships based on shared lived experience; (2) reciprocity and mutuality; (3) validating experiential knowledge; (4) leadership, choice and control; (5) discovering strengths and making connections [20]. We also asked for criteria that applied to (a) recruitment of peer workers, (b) training of peer workers, (c) delivery of peer support, (d) supervision and support for peer workers, (e) organisational support for the peer worker role.

Our previous research about peer support had indicated that different stakeholders could have very different attitudes towards what was important about peer support [21]. We, therefore, took a ‘360°’ approach in assessing fidelity and asked for criteria that might be relevant to peer workers, the people they supported (referred to hereafter as supported peers), peer workers’ supervisors or coordinators and the clinical staff or mental health workers they worked alongside, as well as items that might refer to documentation about the peer worker role (including peer worker role descriptions, training materials, service specifications and organisational policy documents).

We combined similar criteria suggested by both workshops, refined wording and grouped criteria under our five principles (the principles became the domains of the index). We operationalised criteria as items in the fidelity index by writing simple statements for each criterion, to be rated on a Likert scale, as indicators of adherence to the criterion. Where a criterion was relevant to more than one stakeholder (e.g. peer workers and the people they supported), or might also be assessed through documentary evidence, we wrote a statement for each source of data. As a result, some criteria were assessed through multiple items. The index was split into two sections, Setup and Delivery, so that we could assess fidelity to principles in the setting up of a new peer worker intervention and in the ongoing delivery of peer support. Items that assessed the recruitment and training of peer workers, and some aspects of organisational support for the peer worker role constituted the Setup section of the index; items that assessed delivery of peer support, supervision and support for peer workers and other aspects of organisational support constituted the Delivery section.

### Operationalising the index

We used semi-structured interviews and a researcher-rated approach to operationalise the index, rather than inviting respondents to self-rate each item using a Likert scale. This was because researchers working on other aspects of our peer support research programme [18] reported that most research participants were generally positively disposed to peer support and that their initial responses to research questions was often positive, whereas, they might give more nuanced or critical answers when questioned in more depth. We felt as a result that a more open interview schedule would allow these more nuanced responses to emerge, avoiding potential attitudinal bias and increasing the validity of the index, although this would mean that we would need to test the inter-rater reliability of the index. As such, for each of the four stakeholder groups, we wrote semi-structured interview schedules designed to elicit data about each item that applied to that group. We produced a set of guidelines for researchers for undertaking the interviews and for rating responses using 5-point Likert scales. Guidance also covered collecting and rating data from documents.

### Preliminary testing and modifying the index

Interviews (at least one for each stakeholder group) were completed by telephone with 17 services that employed peer workers in one-to-one peer support roles. Services were in the statutory and not-for-profit sectors, inpatient and community mental health service settings, and with people with a range of different mental health diagnoses and experiences. All interviews were undertaken by service user researchers—researchers working from a perspective of having used mental health services—whose insight into conducting interviews and rating data was key to modifying the index. Documentary data were collected for each service, as listed above, as available. All interviews and documents were rated by the researcher collecting the evidence. Interviews were audio recorded and double rated, where we had a complete set for a service (at least one interview from each of the four stakeholder groups). Interviews also elicited feedback about the relevance of the schedule to respondents’ experiences of peer support. Document sets were double rated where these were complete enough to allow all items to be rated.

Ratings of each item were assessed for spread of responses and inter-rater reliability (IRR), calculated using intra-class correlation coefficients (ICCs). As two researchers from a pool of five completed ratings for each double-rated item a two-way random effects model was used to calculate the ICCs, assuming absolute agreement. We used the criteria given by Cicchetti [22] to interpret the acceptability of the estimated ICC’s; poor < 0.40, 0.40—fair—0.59, 0.60—good—0.74, 0.75 + excellent.

Items were retained where spread of response was good (responses were given in at least three of the five Likert categories) and where IRR was at least good. Items were dropped from the schedule where spread of responses was poor (100% of responses were in either the lower or upper two categories) or where ICC was below 0.30. However, we were mindful in dropping items to ensure that the index still adequately incorporated the range of content within each domain. We met as a team to discuss content validity of the index with reference to the principles of peer support framework [20] and, where IRR was intermediate (0.30 < ICC < 0.59) and where the team felt it was important to retain the item to ensure that domains were adequately covered, anchors were developed to enable more reliable rating of Likert statements for those items. Anchors were based on participant feedback on interviews and on researchers’ experiences of rating responses. Minor changes to schedule wording were made, based on participant feedback and researcher experience of undertaking the interviews, so that elicited data might be more focused on criteria being rated. So that a consistent approach could be applied across the index and to improve reliability, anchors were then developed for all retained questions, with short guidance notes as necessary. Researchers reported that it had proved difficult to define anchors for the ‘slightly disagree’ and ‘slightly agree’ points on a five-point scale and so we moved to a three-point scale for all items.

### Retesting the modified index

We selected 10 sets of interviews from the original 17 for re-testing; those which had a full set of sources (supported peer, peer worker, and so on). We conducted interviews, using the modified schedule, with seven additional services providing peer support in our ongoing trial [18]. All 17 sets of interviews and documents were double rated using the new guidance and anchors. Researchers were assigned to rate and re-rate interviews and documents so that no researcher rated data they had rated in preliminary testing. In this modified version, analysis was conducted to establish the IRR and internal consistency (IC) of domain and total scores (all items had sufficient spread of response). IRR was assessed as described above. IC was assessed by Cronbach’s alpha statistics, and is considered acceptable if >  = 0.7. Median, lower quartile (LQ) and upper quartile (UQ) domain and total scores are also reported. Further modifications were then carried out to produce a reliable and valid tool, as described below.

## Results

As a result of the workshops we identified 39 fidelity criteria. A total of 10 criteria applied to principle 1, eight to principle 2, seven to principle 3, seven to principle 4 and seven to principle 5. We arranged the index in two parts: set-up (18 criteria) and delivery (21 criteria). Several criteria were rated using multiple sources, giving a total of 103 items across all sources. There were 49 items in the set-up part of the index and 54 items concerning delivery of peer support.

**Fig. 1:**
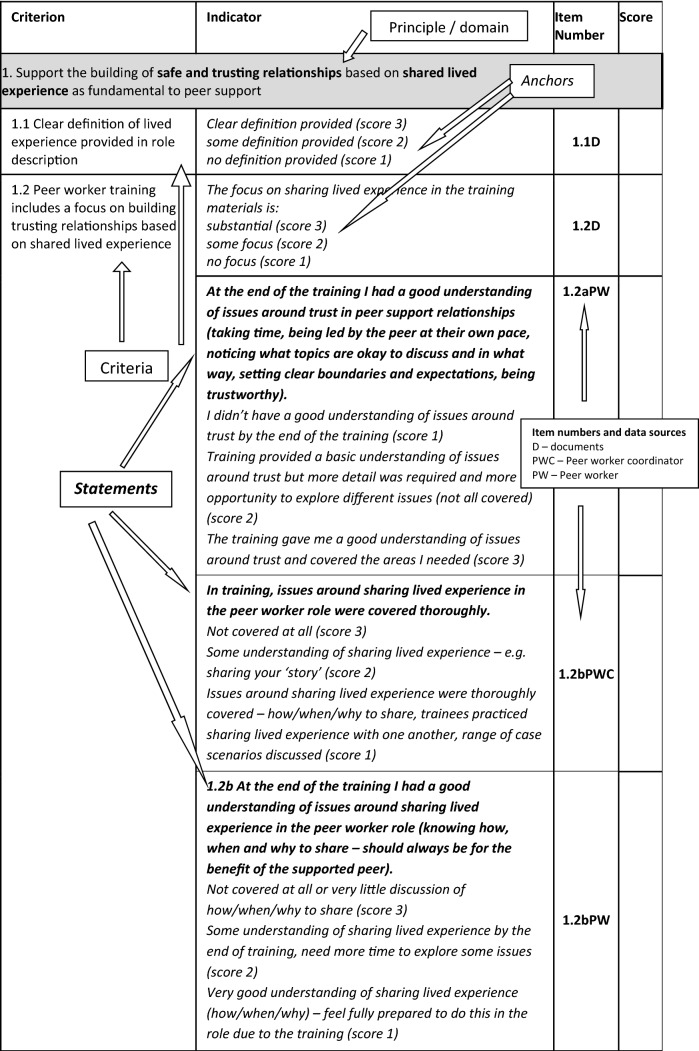
Example of fidelity index structure

Eleven items in the set-up part of the index were rated from documentary data. Thirty-six items were rated from interviews with peer workers (17 set-up and 19 delivery), 32 items from interviews with peer worker coordinators (19 set-up and 13 delivery) and six items from interview with mental health workers (2 set-up and 4 delivery). Eighteen items were rated from interviews with supported peers, all from the delivery part of the index. An example from domain 1 of the draft index covering set-up of the peer worker role, detailing criteria, items, statements, data sources and anchors, is given in Fig. 1 below.

### Preliminary testing

In preliminary testing, 57 interviews from 17 services were completed with 42 interviews double rated, as shown in Table 1 below. All interviews were used in analysis of spread of responses and for feedback on the interview and rating processes. Documents from 14 services were collected with nine document sets sufficiently complete to be double rated. Spread of responses for ratings of each item is given in online Supplementary Tables S1 and S2. ICC statistics as a measure of inter-rater reliability, for each double-rated data source, are also given in Tables S1 and S2. Also indicated are which items were dropped from the index, which retained and which modified.

**Table 1:** Sources of data for preliminary testing

	1st rating	2nd rating
Documents	14	9
Supported peer	12	12
Peer worker	16	12
Peer worker coordinator	14	10
Mental health worker	15	8

All items for mental health workers were dropped (and therefore the mental health worker interview schedule dropped). This was in part because spread of ratings of responses to these items was uneven—responses were more likely to be rated as positive—and in part because IRR scores for these items were poor. Researchers reported that it was sometimes hard to identify a mental health worker who had had sufficient contact with peer support to be able to answer questions meaningfully, and that some mental health workers perhaps wanted the service to be seen in a positive light. As such we felt that these items did not contribute to a reliable assessment of fidelity. As noted above, in dropping the mental health worker schedule we checked that criteria explored in these items were covered in questions asked of other stakeholder groups (and that the overall content validity of the index was maintained).

Eleven items in the set-up section were deleted, nine due to poor IRR (one of which was a mental health worker interview question), one due to high rate of non-response and one further mental health worker question. Eleven further items had low IRR, but were considered key concepts so questions were reworded and anchors developed. One further question was reworded for clarity. One item—about mutuality and reciprocity—was split into two separate items as researchers found responses hard to rate as a single item. In the Delivery section 21 items were deleted, 17 due to poor IRR and four because they were asked of mental health workers. A new item was created for criterion 1.2, to be rated using data collected in the supported peer interview, as all other items for that criterion were deleted and the team felt that there were sufficient data in the SP interview to reliably rate the criterion. Questions for 19 items were reworded for clarity.

### Retesting

For the retesting of the revised index, data from 17 services were rated for the Setup section, 14 for the Delivery section. Three of the additional services had not been operating long enough for the Delivery of their service to be assessed. In the revised index 39 items were rated in the Setup section, 13 from interviews with peer worker coordinators, 15 with peer workers, and 11 from documents. In the Delivery section 34 items were rated, nine from interviews with peer worker coordinators, 11 with peer workers and 14 with supported peers.

Descriptive statistics (Table 2) indicate that there is a greater spread of responses in the Setup section of the index. The spread of response is very low in the Delivery section with median scores tending to be closer to the UQ than the LQ.

**Table 2:** Descriptive, inter-rater reliability and internal consistency statistics for revised fidelity index

Principle	# items	Median	LQ, UQ	ICC	IC
Setup, *n* = 17					
Relationships based on shared lived experience	10	22	17–25	0.73 (0.39, 0.89	0.88
Mutuality and reciprocity	11	24	17–28	0.77 (0.47, 0.91)	0.89
Validating of experiential knowledge	2	5	4–6	0.71 (0.36, 0.88)	− 0.13
Leadership, choice and control	5	12	9–13	0.62 (0.20, 0.84)	0.66
Discovering strengths and making connections	11	24	17–30	0.85 (0.63, 0.94)	0.94
Total setup	39	87	58–97	0.80 (0.53, 0.92)	0.97
Delivery, *n* = 14					
Relationships based on shared lived experience	12	28	25–30	0.48 (− 0.02, 0.80)	0.70
Mutuality and reciprocity	10	19	18–22	0.52 (− 0.01, 0.82)	0.51
Validating of experiential knowledge	3	6	4–7	0.20 (− 0.35, 0.64)	0.36
Leadership, choice and control	4	9	7–10	0.75 (0.37, 0.91)	0.53
Discovering strengths and making connections	5	10.5	9–13	0.58 (0.10, 0.84)	0.38
Total	34	71	68–72	0.53 (0.04, 0.82)	0.89

*LQ* lower quartile, *UQ* upper quartile, *ICC* intra-class correlation coefficient, *IC* internal consistency

ICC statistics for the Setup section (Table 2) of the index are categorised as ‘good’ or ‘excellent’ (all > 0.6) for the five domains and total score. IC was very low for the third domain perhaps because the domain has only two items, from different sources, peer workers and documentary evidence. The IRR for the peer worker item was low (ICC = 0.37) so when this item was deleted this left only the documentary item which had an ICC of 0.75. The IC for the fourth domain just failed to reach the acceptable threshold.

For the Delivery section the reliability statistics were, on the whole, not acceptable. Only one domain reached an acceptable level of ICC. Internal consistency was acceptable for the first domain and the total score.

### Final modification

It was decided to repeat an assessment of the IRR of the individual items in the delivery section (online Supplementary Table S3). In this, further modification of the Delivery section items with more than two missing observations or an ICC < 0.4 (poor) were deleted. Two supported peer items were deleted, one for peer worker coordinators and six for peer workers.

Only one item was removed from the Setup section in Table 3 causing small changes to the total score. In the modified Delivery section all ICCs reach the ‘good’ categorisation for IRR apart from, marginally, the fifth domain. Only the total score had an acceptable IC, 0.81. The ICC and IC estimates were also calculated for the overall index. The third domain was removed as it retained only a single item. ICCs were all 0.67 or above and IC estimates all acceptable except for the Leadership, choice and control domain.

**Table 3:** Descriptive, inter-rater reliability and internal consistency statistics for modified revised fidelity index

Principle	# data sources	Median	LQ, UQ	ICC	IC
Setup, *n* = 17					
Relationships based on shared lived experience	10	22	17–25	0.75 (0.43, 0.90)	0.88
Mutuality and reciprocity	11	24	17–28	0.77 (0.47, 0.91)	0.89
Leadership, choice and control	5	12	9–13	0.62 (0.20, 0.84)	0.66
Discovering strengths and making connections	11	24	17–30	0.85 (0.63, 0.94)	0.94
Total setup	38	77	51–90.5	0.82 (0.58, 0.93)	0.97
Delivery, *n* = 14					
Relationships based on shared lived experience	8	19	16.3–20.3	0.72 (0.33, 0.90)	0.61
Mutuality and reciprocity	8	15	13.8–16.5	0.65 (0.22, 0.87)	0.48
Leadership, choice and control	4	9	7–10	0.75 (0.37, 0.91)	0.53
Discovering strengths and making connections	5	10.5	8.8–13	0.58 (0.10, 0.84)	0.38
Total	25	52.5	48.3–55	0.69 (0.29, 0.89)	0.81
Combined index, *n* = 14					
Relationships based on shared lived experience	18	38	32–43.3	0.74 (0.38, 0.91)	0.81
Mutuality and reciprocity	19	36.5	30.5–42.8	0.70 (0.31, 0.89)	0.82
Leadership, choice and control	9	20	14.8–23	0.67 (0.23, 0.88)	0.23
Discovering strengths and making connections	16	29.5	25.5–39.3	0.78 (0.44, 0.92)	0.90
Total	62	122.5	100.8–145.8	0.75 (0.41, 0.91)	0.95

*LQ* lower quartile, *UQ* upper quartile, *ICC* intra-class correlation coefficient, *IC* internal consistency

## Discussion

The final version of the principle-based fidelity index for peer support interventions in mental health services developed in this study demonstrated acceptable psychometric properties when tested in a range of different peer support services. The combined index—incorporating a measure of the fidelity of both the setup and delivery of peer support—demonstrated good inter-rater reliability across four domains: sense of connection based on shared lived experience; relationships characterised by mutuality and reciprocity; peers’ ability to exercise leadership, choice and control in the way peer support takes place; peer support focused on discovering individual strengths and making connections to community. Internal consistency was good overall and in three of the four domains. We suggest that internal consistency might be low in the ‘leadership, choice and control’ domain as items explored these constructs at a range of levels, personal, inter-personal and organisational, although this would warrant further investigation. The psychometric properties of the setup portion of the index are good overall and across all four domains, and are good overall for the delivery portion of the index. We removed the ‘validating experiential knowledge’ domain from the index. We reflect that the constructs articulated here were perhaps too abstract to be reliably explored and rated using the index. We note that removing this domain potentially impacts on the overall content validity of the index in relation to the principles of peer support framework [20]. However, we suggest that the importance of shared, experiential learning in the peer support relationship [2] is sufficiently covered by the criteria rated in the ‘mutuality and reciprocity’ domain.

### Strengths and weaknesses

This was a thorough, coherent process of fidelity index development, assessing fidelity in a broader range of domains than structural aspects of intervention delivery alone and informed by the same theoretical framework that underpinned the intervention [9]. We report the full cycle of fidelity index development, including identification of fidelity items, operationalisation and robust statistical testing of the index [19].

We acknowledge that changes made to interview schedules during index development, although minor, could not be applied retrospectively to the first set of interviews when we retested the index. However, those changes were designed to ensure that data elicited were more clearly focused on the criteria to be rated and as such subsequent results are perhaps more likely to have underestimated reliability. Heterogeneity in the peer support interventions we included in testing is also likely to have impacted reliability of observed ratings; i.e. if we had only tested the index in, for example, peer support provided in community mental health services in the statutory sector, we might have observed higher IRR. However, that same heterogeneity enables us to suggest that the validity of our index, as indicated above, extends to a range of peer support interventions across clinical settings and organisational contexts. The inclusion, in our expert panel, of people with expertise in peer support in a wide range of contexts—and especially the role the panel played in generating items for the index—hopefully supports that extended validity. That the measurement of fidelity is based on the responses of people receiving, providing and supervising peer support potentially lends additional validity to the index.

We note that further testing of the internal structure of the index, for example through a confirmatory factor analysis, might provide additional evidence of the validity of the index. This might usefully be undertaken were the index to be applied to a larger sample in future studies. Similarly, to establish construct validity for the index, it would be necessary to explore the relationship between fidelity scores for groups of participants or peer support services, and outcomes that might be expected to be associated with fidelity. Again, this might be undertaken as part of further research to fully establish the psychometric properties of the index. On balance we feel that using the index offers a good overall measure of principle-based fidelity of peer support interventions in mental health services, and across four specific domains related to those principles [20].

### Implications

This study adds to existing contributions in assessing the fidelity of peer support interventions in mental health services [15–17] and makes a novel contribution in that our index assesses the fidelity of peer support interventions to principles that characterise the peer support relationship as distinct from clinician-patient relationships in mental health services. The index provides a means of measuring the extent to which peer support is being delivered in a way that is demonstrably different from care as usual, and is therefore, likely to help in the interpretation of evaluations of peer support interventions, addressing limitations to the evidence base for peer support hitherto identified [5, 6].

The index also enables fidelity to principles in the setting up of a new peer support initiative to be measured, as well as the ongoing delivery of an existing peer support intervention. Given that a number of items relate to the training of peer workers [9], the Set-up section of the index might also be used following the training of a new cohort of peer workers to an existing initiative. The index also offers service commissioners and providers a way of assessing and ensuring that the distinctive qualities of peer support are supported and retained, especially in highly regulated health service environments [21, 23], reflecting calls made internationally for standards to be applied to the delivery of peer support in mental health services [14]. The index is timely, therefore, as mental health workforce policy stipulates the introduction of large numbers of new peer workers into mental health services [24, 25], providing an opportunity to ensure that these developments are informed by the best available evidence.

## Supplementary Information

Below is the link to the electronic supplementary material.

Supplementary file1 (DOCX 28 KB)

Supplementary file2 (DOCX 29 KB)

Supplementary file3 (DOC 85 KB)

## Data Availability

All data and materials used in this study will be made available on reasonable request to the corresponding author.

## Code availability

All statistical analysis was conducted in IBM SPSS Statistics v25.

## References

[1] Gillard S, Gibson SL, Holley J, Lucock M (2015). Developing a change model for peer worker interventions in mental health services: a qualitative research study. Epidemiol Psychiatri Sci.

[2] Mead S, Filson B (2017). Mutuality and shared power as an alternative to coercion and force. Mental Health Soc Incl.

[3] Oborn E, Barrett M, Gibson S, Gillard S (2019). Knowledge and expertise in care practices: the role of the peer worker in mental health teams. Sociol Health Illn.

[4] Gillard S, Edwards C, Gibson SL, Owen K, Wright C (2013). Introducing peer worker roles into UK mental health service teams: a qualitative analysis of the organisational benefits and challenges. BMC Health Serv Res.

[5] Pitt V, Lowe D, Hill S, Prictor M, Hetrick SE, Ryan R, Berends L (2013). Consumer-providers of care for adult clients of statutory mental health services. Cochrane Database Syst Rev.

[6] Lloyd-Evans B, Mayo-Wilson E, Harrison B, Istead H, Brown E, Pilling S, Johnson S, Kendall T (2014). A systematic review and meta-analysis of randomised controlled trials of peer support for people with severe mental illness. BMC Psychiatry.

[7] King A, Simmons M (2018). A systematic review of the attributes and outcomes of peer work and guidelines for reporting studies of peer interventions. Psychiatr Serv.

[8] Moncher FJ, Prinz RJ (1991). Treatment fidelity in outcome studies. Clin Psychol Rev.

[9] Gearing RE, El-Bassel N, Ghesquiere A, Baldwin S, Gillies J, Ngeow E (2011). Major ingredients of fidelity: a review and scientific guide to improving quality of intervention research implementation. Clin Psychol Rev.

[10] Coleman K, Austin BT, Brach C, Wagner EH (2009). Evidence on the chronic care model in the new millennium. Health Aff.

[11] Hasson H, Blomberg S, Dunér A (2012). Fidelity and moderating factors in complex interventions: a case study of a continuum of care program for frail elderly people in health and social care. Implement Sci.

[12] Muntinga ME, Van Leeuwen KM, Schellevis FG, Nijpels G, Jansen A (2015). From concept to content: assessing the implementation fidelity of a chronic care model for frail, older people who live at home. BMC Health Serv Res.

[13] O’Hagan M, McKee H, Priest R (2009). Consumer survivor initiatives in Ontario: building for an equitable future.

[14] Stratford A, Halpin M, Phillips K, Skerritt F, Beales A, Cheng V (2017). The growth of peer support: an international charter. J Mental Health.

[15] Ostrow L, Croft B (2014) Toolkit for Evaluating Peer Respites. (https://www.hsri.org/publication/peer-respite-toolkit). Accessed 29 June 2020.

[16] Garber-Epstein P, Zisman-Ilani Y, Levine S, Roe D (2013). Comparative impact of professional mental health background on ratings of consumer outcome and fidelity in an illness management and recovery program. Psychiatr Rehabil J.

[17] Chinman M, McCarthy S, Mitchell-Miland C, Daniels K, Youk A, Edelen M (2016). Early stages of development of a peer specialist fidelity measure. Psychiatr Rehabil J.

[18] Gillard S, Bremner S, Foster R, Gibson SL, Goldsmith L, Healey A, Lucock M, Marks J, Morshead R, Patel A, Priebe S (2020) Peer support for discharge from inpatient to community mental health services: Study protocol clinical trial (SPIRIT Compliant). Medicine 99(10):e19192 10.1097/MD.0000000000019192 PMC7478470 32150057

[19] Mowbray CT, Holter MC, Teague GB, Bybee D (2003). Fidelity criteria: development, measurement, and validation. Am J Eval.

[20] Gillard S, Foster R, Gibson S, Goldsmith L, Marks J, White S (2017) Describing a principles-based approach to developing and evaluating peer worker roles as peer support moves into mainstream mental health services. Ment Health Soc Incl 21:133–143

[21] Gillard S, Holley J, Gibson S, Larsen J, Lucock M, Oborn E, Rinaldi M, Stamou E (2015) Introducing new peer worker roles into mental health services in England: comparative case study research across a range of organisational contexts. Adm Policy Ment Health Ment Health Serv Res 42:682–694 10.1007/s10488-014-0603-z 25331447

[22] Cicchetti DV (1994). Guidelines, criteria, and rules of thumb for evaluating normed and standardized assessment instruments in psychology. Psychol Assess.

[23] Stewart S, Watson S, Montague R, Stevenson C (2008). “Set up to fail?” Consumer participation in the mental health service system. Aust Psychiatry.

[24] Council of Australia Governments (2017) The Fifth National Mental Health and Suicide Prevention Plan. Canberra: Council of Australia Governments Health Council. (http://www.coaghealthcouncil.gov.au/Portals/0/Fifth%20National%20Mental%20Health%20and%20Suicide%20Prevention%20Plan.pdf). Accessed 17 June 2020.

[25] Health Education England (2017) Stepping forward to 2020/21: The mental health workforce plan for England. Leeds: Health Education England. (https://www.basw.co.uk/system/files/resources/basw_62959-3_0.pdf). Accessed 17 June 2020.

