# Supplementary material for: Developing and testing a principle-based fidelity index for peer support in mental health services
Source: Soc Psychiatry Psychiatr Epidemiol. 2021 Feb 19;56(10):1903–11. doi: 10.1007/s00127-021-02038-4 (PMC8429155; doi:10.1007/s00127-021-02038-4)
Supplement: Supplementary file 1 — Supplementary file1 (DOCX 28 KB) [file 127_2021_2038_MOESM1_ESM.docx]

Supplementary Table S1: Preliminary testing - Setup

| **Item** | **Source** | **1** | **2** | **3** | **4** | **5** | **n** | **missing** | **ICC (95% CI)** |  |
| --- | --- | --- | --- | --- | --- | --- | --- | --- | --- | --- |
| **1.1** | Doc | 4 | 0 | 2 | 0 | 8 | 14 | 0 | 0.78 (0.29, 0.95) | R |
| **1.2** | Doc | 4 | 0 | 3 | 0 | 7 | 14 | 0 | 0.80 (0.1, 0.98) | R |
| **1.2a** | PWC | 0 | 1 | 3 | 4 | 7 | 15 | 0 | -0.50 (-0.97, 0.24) | D |
| **1.2a** | PW | 4 | 1 | 1 | 4 | 6 | 16 | 0 | 0.71 (0.24, 0.91) | R |
| **1.2b** | PWC | 1 | 0 | 2 | 5 | 7 | 15 | 0 | 0.69 (0.12, 0.91) | W |
| **1.2b** | PW | 3 | 3 | 1 | 3 | 6 | 16 | 0 | 0.71 (0.28, 0.91) | R |
| **1.3** | Doc | 3 | 0 | 2 | 2 | 7 | 14 | 0 | 0.00 (-0.81, 0.81) | R |
| **1.3** | PWC | 0 | 0 | 0 | 7 | 8 | 15 | 0 | -0.41 (-0.95, 0.34) | W |
| **1.3** | PW | 1 | 2 | 1 | 4 | 8 | 16 | 0 | 0.30 (-0.36, 0.74) | R |
| **1.4** | PWC | 2 | 2 | 3 | 2 | 3 | 12 | 3 | 0.52 (-0.34, 0.90) | W |
| **1.4** | PW | 2 | 2 | 4 | 3 | 1 | 12 | 4 | 0.54 (-0.13, 0.90) | W |
| **1.4** | MHW | 0 | 0 | 3 | 6 | 7 | 16 | 0 | 0.23 (-0.17, 0.69) | D |
| **2.1** | Doc | 6 | 0 | 1 | 0 | 7 | 14 | 0 | 0.96 (0.74, 1.0) | R |
| **2.1a** | PWC | 0 | 1 | 5 | 3 | 6 | 15 | 0 | -0.39 (-0.94, 0.38) | R |
| **2.1a** | PW | 2 | 1 | 4 | 3 | 5 | 15 | 1 | 0.75 (0.31, 0.93) | R |
| **2.1b** | PWC | 0 | 0 | 4 | 3 | 8 | 15 | 0 | -0.29 (-0.84, 0.44) | R |
| **2.1b** | PW | 1 | 2 | 5 | 2 | 6 | 16 | 0 | 0.35 (-0.29, 0.76) | R |
| **2.2** | Doc | 7 | 0 | 1 | 0 | 6 | 14 | 0 | 0.67 (0.03, 0.93) | R |
| **2.3** | PWC | 0 | 0 | 2 | 2 | 11 | 15 | 0 | 0.32 (-0.25, 0.77) | R |
| **2.3** | PW | 1 | 1 | 7 | 2 | 4 | 15 | 1 | 0.34 (-0.20, 0.75) | D |
| **2.4** | Doc | 5 | 0 | 1 | 1 | 7 | 14 | 0 | 0.60 (-0.17, 0.93) | R |
| **2.4** | PWC | 0 | 3 | 1 | 5 | 6 | 15 | 0 | 0.39 (-0.35, 0.81) | W |
| **2.4** | PW | 2 | 2 | 2 | 4 | 6 | 16 | 0 | 0.47 (-0.10, 0.81) | R |
| **3.1** | Doc | 4 | 0 | 4 | 0 | 6 | 14 | 0 | 0.67 (0.02, 0.92) | R |
| **3.3** | PWC | 0 | 0 | 2 | 6 | 7 | 15 | 0 | 0.08 (-0.54, 0.67) | D |
| **3.3** | PW | 2 | 1 | 1 | 2 | 9 | 15 | 1 | 0.07 (-62, 0.64) | R |
| **4.1** | Doc | 5 | 0 | 2 | 0 | 6 | 13 | 1 | 0.92 (0.43, 1.0) | R |
| **4.1** | PWC | 0 | 0 | 6 | 3 | 6 | 15 | 0 | 0.29 (-0.44, 0.77) | D |
| **4.1** | PW | 2 | 2 | 1 | 2 | 9 | 16 | 0 | 0.34 (-0.28, 0.75) | W |
| **4.2** | PWC | 2 | 2 | 1 | 3 | 3 | 11 | 4 | 0.33 (-0.43, 0.89) | S |
| **4.2** | PW | 3 | 2 | 2 | 2 | 1 | 10 | 6 | 0.73 (-0.02, 0.96) | D |
| **4.2** | MHW | 5 | 0 | 3 | 2 | 2 | 12 | 4 | -0.11 (-1.0, 0.70) | D |
| **4.3a** | PWC | 1 | 3 | 2 | 3 | 6 | 15 | 0 | 0.40 (-0.34, 0.81) | R |
| **4.3b** | PWC | 0 | 1 | 5 | 3 | 6 | 15 | 0 | -0.18 (-0.75, 0.49) | W |
| **5.1** | PWC | 0 | 2 | 1 | 4 | 8 | 15 | 0 | 0.41 (-0.23, 0.81) | D |
| **5.1** | PW | 1 | 2 | 2 | 3 | 8 | 16 | 0 | 0.70 (0.23, 0.90) | R |
| **5.2** | Doc | 5 | 0 | 2 | 1 | 6 | 14 | 0 | 0.75 (0.02, 0.99) | R |
| **5.2a** | PWC | 1 | 2 | 1 | 5 | 6 | 15 | 0 | -0.36 (-0.94, 0.38) | W |
| **5.2a** | PW | 2 | 2 | 0 | 7 | 5 | 16 | 0 | 0.73 (0.27, 0.92) | R |
| **5.2b** | PWC | 3 | 1 | 3 | 3 | 5 | 15 | 0 | 0.50 (-0.17, 0.85) | D |
| **5.2b** | PW | 4 | 3 | 2 | 5 | 2 | 16 | 0 | 0.53 (-0.08, 0.84) | D |
| **5.3a** | Doc | 6 | 3 | 2 | 2 | 1 | 14 | 0 | 0.90 (0.18, 0.99) | R |
| **5.3b** | Doc | 7 | 3 | 3 | 0 | 1 | 14 | 0 | 0.71 (-0.08, 0.96) | R |
| **5.3c** | PWC | 1 | 0 | 3 | 4 | 7 | 15 | 0 | -0.28 (-0.76, 0.39) | W |
| **5.3c** | PW | 7 | 3 | 1 | 1 | 4 | 16 | 0 | 0.52 (0.00, 0.83) | W |
| **5.3d** | PWC | 1 | 0 | 1 | 5 | 8 | 15 | 0 | -0.05 (-0.43, 0.49) | D |
| **5.3d** | PW | 4 | 3 | 1 | 6 | 2 | 16 | 0 | 0.03 (-0.59, 0.59) | W |
| **5.4** | PWC | 2 | 0 | 3 | 3 | 7 | 15 | 0 | 0.44 (-0.32, 0.84) | W |
| **5.4** | PW | 3 | 2 | 5 | 3 | 3 | 16 | 0 | 0.38 (-0.25, 0.78) | R |

Key: Doc = document; PW = Peer Worker; PWC = Peer Worker Coordinator; MHW = mental health worker; ICC= intra-class correlation coefficient; CI = confidence interval; R = item retained; W = item wording modified; D = item deleted; S = item split into two new items
