# Supplementary material for: Developing and testing a principle-based fidelity index for peer support in mental health services
Source: Soc Psychiatry Psychiatr Epidemiol. 2021 Feb 19;56(10):1903–11. doi: 10.1007/s00127-021-02038-4 (PMC8429155; doi:10.1007/s00127-021-02038-4)
Supplement: Supplementary file 2 — Supplementary file2 (DOCX 29 KB) [file 127_2021_2038_MOESM2_ESM.docx]

**Supplementary Table S2: Preliminary testing - Delivery**

|  |  | **1** | **2** | **3** | **4** | **5** | **n** | **missing** | **ICC (95% CI)** |  |
| --- | --- | --- | --- | --- | --- | --- | --- | --- | --- | --- |
| **1.1** | SP | 1 | 1 | 3 | 2 | 5 | 12 | 0 | 0.39 (-0.35, 0.81) | R |
| **1.1** | PW | 0 | 2 | 2 | 4 | 8 | 16 | 0 | 0.22 (-0.47, 0.72) | W |
| **1.2** | PWC | 0 | 1 | 1 | 6 | 6 | 14 | 0 | -0.10 (-0.77, 0.59) | D |
| **1.2** | PW | 1 | 5 | 0 | 1 | 9 | 16 | 0 | 0.09 (-0.60, 0.65) | D |
| **1.3** | SP | 1 | 0 | 2 | 2 | 7 | 12 | 0 | 0.75 (0.30, 0.93) | R |
| **1.4** | PWC | 1 | 0 | 2 | 1 | 10 | 14 | 0 | -0.20 (-0.84, 0.50) | W |
| **1.4** | PW | 1 | 4 | 1 | 3 | 7 | 16 | 0 | 0.47 (-0.10, 0.81) | W |
| **1.5** | SP | 2 | 0 | 1 | 2 | 4 | 9 | 3 | 0.74 (0.09, 0.95) | W |
| **1.5** | PWC | 1 | 1 | 4 | 1 | 7 | 14 | 0 | -0.17 (-0.83, 0.52) | D |
| **1.5** | PW | 1 | 1 | 4 | 3 | 7 | 16 | 0 | 0.43 (-0.17, 0.80) | W |
| **1.5** | MHW | 1 | 2 | 3 | 1 | 7 | 14 | 1 | -0.04 (-0.78, 0.64) | D |
| **1.6a** | PWC | 0 | 4 | 1 | 3 | 6 | 14 | 0 | -0.39 (-0.88, .0.39) | W |
| **1.6a** | PW | 1 | 2 | 2 | 2 | 9 | 16 | 0 | 0.58 (0.01, 0.86) | W |
| **1.6b** | PWC | 0 | 2 | 2 | 1 | 9 | 14 | 0 | 0.63 (0.01, 0.89) | R |
| **1.6b** | PW | 2 | 3 | 3 | 2 | 6 | 16 | 0 | 0.50 (-0.13, 0.84) | R |
| **2.1a** | SP | 0 | 0 | 2 | 1 | 9 | 12 | 0 | 0.31 (-0.39, 0.77) | W |
| **2.1a** | PW | 0 | 1 | 3 | 3 | 9 | 16 | 0 | 0.11 (-0.50, 0.63) | D |
| **2.1b** | SP | 2 | 0 | 1 | 0 | 9 | 12 | 0 | 0.47 (-0.22, 0.84) | R |
| **2.1b** | PW | 2 | 2 | 3 | 1 | 8 | 16 | 0 | 0.54 (-0.05, 0.84) | R |
| **2.2a** | SP | 0 | 1 | 2 | 2 | 7 | 12 | 0 | -0.12 (-0.81, 0.53) | W |
| **2.2b** | SP | 7 | 1 | 1 | 0 | 2 | 11 | 1 | 0.88 (0.44, 0.98) | R |
| **2.2b** | PW | 4 | 2 | 1 | 2 | 4 | 13 | 2 | 0.13 (-0.57, 0.71) | D |
| **2.2c** | SP | 3 | 1 | 2 | 0 | 2 | 8 | 4 | 0.38 (-1.95, 0.98) | D |
| **2.2c** | PW | 1 | 2 | 2 | 4 | 7 | 16 | 0 | 0.34 (-0.28, 0.77) | D |
| **2.3a** | PWC | 0 | 3 | 2 | 4 | 5 | 14 | 0 | 0.28 (-0.41, 0.76) | D |
| **2.3a** | PW | 1 | 4 | 4 | 2 | 5 | 16 | 0 | 0.54 (0.04, 0.84) | W |
| **2.3b** | PWC | 3 | 2 | 3 | 4 | 1 | 13 | 1 | 0.27 (-0.51, 0.78) | W |
| **2.3b** | PW | 8 | 3 | 1 | 3 | 1 | 16 | 0 | 0.17 (-0.49, 0.67) | W |
| **2.4** | PWC | 3 | 4 | 4 | 1 | 2 | 14 | 0 | 0.50 (-0.11, 0.84) | R |
| **2.4** | MHW | 3 | 6 | 1 | 3 | 3 | 16 | 0 | 0.48 (-0.07, 0.82) | D |
| **3.1** | PW | 2 | 0 | 5 | 3 | 3 | 13 | 3 | 0.50 (-0.35, 0.88) | W |
| **3.1** | MHW | 0 | 1 | 0 | 5 | 10 | 16 | 0 | -0.22 (-0.80, 0.47) | D |
| **3.2a** | SP | 0 | 1 | 1 | 0 | 10 | 12 | 0 | -0.25 (-0.87, 0.49) | W |
| **3.2b** | SP | 2 | 0 | 1 | 1 | 8 | 12 | 0 | 0.07 (-0.72, 0.69) | D |
| **3.3** | SP | 3 | 1 | 3 | 1 | 4 | 12 | 0 | 0.10 (-0.68, 0.70) | D |
| **3.3** | PW | 0 | 0 | 6 | 2 | 8 | 16 | 0 | 0.15 (-0.47, 0.65) | D |
| **3.4** | PW | 0 | 3 | 6 | 0 | 6 | 15 | 1 | 0.50 (-0.08, 0.82) | R |
| **4.1** | SP | 0 | 0 | 3 | 2 | 7 | 12 | 0 | 0.14 (-0.65, 0.73) | W |
| **4.1** | PW | 0 | 1 | 1 | 3 | 11 | 16 | 0 | 0.25 (-0.35, 0.71) | D |
| **4.2a** | PWC | 1 | 0 | 4 | 5 | 4 | 14 | 0 | -0.51 (-1.00, 0.24) | D |
| **4.2b** | PWC | 0 | 1 | 4 | 2 | 6 | 13 | 0 | -0.28 (-0.93, 0.49) | W |
| **4.2c** | PWC | 0 | 0 | 4 | 5 | 3 | 12 | 2 | 0.16 (-0.72, 0.76) | W |
| **4.2c** | MHW | 0 | 2 | 2 | 4 | 6 | 14 | 1 | 0.16 (-0.48, 0.73) | D |
| **4.3** | PW | 0 | 2 | 4 | 2 | 8 | 16 | 0 | 0.17 (-0.51, 0.69) | D |
| **4.3** | PWC | 1 | 1 | 2 | 4 | 6 | 14 | 0 | 0.48 (-0.22, 0.85) | W |
| **4.4** | SP | 0 | 2 | 1 | 1 | 8 | 12 | 0 | 0.39 (-0.34, 0.81) | D |
| **5.1** | PW | 1 | 3 | 4 | 2 | 6 | 16 | 0 | 0.44 (-0.19, 0.80) | W |
| **5.1** | PWC | 0 | 1 | 3 | 4 | 6 | 14 | 0 | 0.04 (-0.60, 0.63) | R |
| **5.2** | SP | 1 | 0 | 1 | 4 | 6 | 12 | 0 | 0.66 (0.03, 0.91) | R |
| **5.2** | PW | 0 | 1 | 2 | 6 | 7 | 16 | 0 | -0.25 (-0.78, 0.41) | D |
| **5.3a** | SP | 0 | 2 | 2 | 1 | 7 | 12 | 0 | 0.91 (0.65, 0.98) | R |
| **5.3b** | SP | 2 | 1 | 4 | 1 | 2 | 10 | 2 | 0.72 (0.14, 0.94) | R |
| **5.3c** | SP | 0 | 1 | 6 | 0 | 4 | 11 | 1 | 0.62 (-0.04, 0.91) | R |
| **5.3d** | SP | 1 | 1 | 1 | 6 | 2 | 11 | 1 | -0.13 (-0.65, 0.56) | D |

Key: SP = supported peer; PW = Peer Worker; PWC = Peer Worker Coordinator; MHW = mental health worker; ICC= intra-class correlation coefficient; CI = confidence interval; R = item retained; W = item wording modified; D = item deleted
