# Supplementary material for: Developing and testing a principle-based fidelity index for peer support in mental health services
Source: Soc Psychiatry Psychiatr Epidemiol. 2021 Feb 19;56(10):1903–11. doi: 10.1007/s00127-021-02038-4 (PMC8429155; doi:10.1007/s00127-021-02038-4)
Supplement: Supplementary file 3 — Supplementary file3 (DOC 85 KB) [file 127_2021_2038_MOESM3_ESM.doc]

Supplementary Table S3: Final modification - Delivery

| **Item** | **Source** | **# missing** | **ICC** |  |
| --- | --- | --- | --- | --- |
| 1.1 | PW | 0 | 0.22 (-0.38, 0.67) | D |
| 1.1 | SP | 0 | 0.65 (0.22, 0.87) | R |
| 1.2 | SP | 0 | 0.75 (0.40, 0.91) | R |
| 1.3 | SP | 0 | 0.70 (0.29, 0.89) | R |
| 1.4 | PWC | 0 | 0.38 (-0.19, 0.75) | D |
| 1.4 | PW | 0 | 0.14 (-0.45, 0.62) | D |
| 1.5 | PW | 0 | 0.48 (-0.04, 0.80) | R |
| 1.5 | SP | 0 | 0.47 (-0.03, 0.79) | R |
| 1.6a | PWC | 0 | 0.40 (-0.17, 0.76) | R |
| 1.6a | PW | 0 | 0.39 (-0.18, 0.75) | D |
| 1.6b | PWC | 0 | 0.53 (0.00, 0.83 | R |
| 1.6b | PW | 0 | 0.47 (-0.04, 0.79) | R |
| 2.1a | SP | 0 | 0.66 (0.22, 0.87) | R |
| 2.1b | PW | 0 | -0.13 (-0.64, 0.43) | D |
| 2.1b | SP | 0 | 0.40 (-0.10, 0.76) | R |
| 2.2 | SP | 0 | 0.78 (0.46, 0.92) | R |
| 2.2a | SP | 0 | 0.02 (-0.49, 0.53) | D |
| 2.2c | SP | 0 | 0.44 (-0.09, 0.78) | R |
| 2.3a | PW | 0 | 0.48 (-0.02, 0.80) | R |
| 2.3b | PWC | 0 | 0.57 (0.06, 0.84) | R |
| 2.3b | PW | 0 | 0.52 (0.00, 0.82) | R |
| 2.4 | PWC | 0 | 0.74 (0.36, 0.91) | R |
| 3.1 | PW | 6 | 0.53 (-0.24, 0.89) | D |
| 3.2a | SP | 0 | 0.12 (-0.47, 0.61) | D |
| 3.4 | PW | 0 | -0.21 (-0.70, 0.36) | D |
| 4.1 | SP | 0 | 0.72 (0.32, 0.90) | R |
| 4.2b | PWC | 0 | 0.61 (0.15, 0.86) | R |
| 4.2c | PWC | 4 | 0.69 (0.18, 0.91) | R |
| 4.3 | PWC | 0 | 0.57 (0.05, 0.84) | R |
| 5.1 | PWC | 0 | 0.74 (0.36, 0.91) | R |
| 5.1 | PW | 0 | 0.64 (0.18, 0.87) | R |
| 5.3a | SP | 0 | 0.54 (0.02, 0.93) | R |
| 5.3b | SP | 0 | 0.45 (-0.08, 0.78) | R |
| 5.3c | SP | 0 | 0.41 (-0.17, 0.77) | R |

Key: SP = supported peer; PW = Peer Worker; PWC = Peer Worker Coordinator; ICC= intra-class correlation coefficient; R = item retained; D = item deleted
